# COVID-19 impairs oxygen delivery by altering red blood cell hematological, hemorheological, and oxygen transport properties

**DOI:** 10.3389/fphys.2023.1320697

**Published:** 2024-01-03

**Authors:** Stephen C. Rogers, Mary Brummet, Zohreh Safari, Qihong Wang, Tobi Rowden, Tori Boyer, Allan Doctor

**Affiliations:** Divisions of Critical Care Medicine and the Center for Blood Oxygen Transport and Hemostasis, Department of Pediatrics, University of Maryland School of Medicine, Baltimore, MD, United States

**Keywords:** red blood cell, coronavirus disease 2019, oxygen, rheology, osmotic fragility, deformability, aggregation, vasoactivity

## Abstract

**Introduction:** Coronavirus disease 2019 (COVID-19) is characterized by impaired oxygen (O_2_) homeostasis, including O_2_ sensing, uptake, transport/delivery, and consumption. Red blood cells (RBCs) are central to maintaining O_2_ homeostasis and undergo direct exposure to coronavirus *in vivo*. We thus hypothesized that COVID-19 alters RBC properties relevant to O_2_ homeostasis, including the hematological profile, Hb O_2_ transport characteristics, rheology, and the hypoxic vasodilatory (HVD) reflex.

**Methods:** RBCs from 18 hospitalized COVID-19 subjects and 20 healthy controls were analyzed as follows: (i) clinical hematological parameters (complete blood count; hematology analyzer); (ii) O_2_ dissociation curves (p50, Hill number, and Bohr plot; Hemox-Analyzer); (iii) rheological properties (osmotic fragility, deformability, and aggregation; laser-assisted optical rotational cell analyzer (LORRCA) ektacytometry); and (iv) vasoactivity (the RBC HVD; vascular ring bioassay).

**Results:** Compared to age- and gender-matched healthy controls, COVID-19 subjects demonstrated 1) significant hematological differences (increased WBC count—with a higher percentage of neutrophils); RBC distribution width (RDW); and reduced hematocrit (HCT), Hb concentration, mean corpuscular volume (MCV), and mean corpuscular hemoglobin concentration (MCHC); 2) impaired O_2_-carrying capacity and O_2_ capacitance (resulting from anemia) without difference in p50 or Hb–O_2_ cooperativity; 3) compromised regulation of RBC volume (altered osmotic fragility); 4) reduced RBC deformability; 5) accelerated RBC aggregation kinetics; and (6) no change in the RBC HVD reflex.

**Discussion:** When considered collectively, homeostatic compensation for these RBC impairments requires that the cardiac output in the COVID cohort would need to increase by ∼135% to maintain O_2_ delivery similar to that in the control cohort. Additionally, the COVID-19 disease RBC properties were found to be exaggerated in blood-type O hospitalized COVID-19 subjects compared to blood-type A. These data indicate that altered RBC features in hospitalized COVID-19 subjects burden the cardiovascular system to maintain O_2_ delivery homeostasis, which appears exaggerated by blood type (more pronounced with blood-type O) and likely plays a role in disease pathogenesis.

## Introduction

Red blood cells (RBCs), the most abundant cells in the body (Sender et al., 2016), play an essential role in oxygen (O_2_) homeostasis (i.e., O_2_ sensing, uptake, transport, and delivery). Whilst the RBC number and hemoglobin concentration (Hb) define blood O_2_-carrying capacity, homeostatic modulation of Hb–O_2_ affinity ultimately regulates O_2_ capture/release in a manner that stabilizes O_2_ delivery in the setting of reduced O_2_ availability (i.e., hypoxia and anemia) or increased consumption (i.e., stress and disease). This effect is achieved via the production of allosteric effectors (i.e., 2,3 DPG and ATP) and adjustment of the RBC internal milieu (i.e., pH and anion concentration) in response to external stimuli (i.e., temperature and CO_2_ tension). In addition to modulating Hb–O_2_ affinity, RBCs play a direct but less well-appreciated role in O_2_ delivery homeostasis by regulating blood flow itself. This includes both active signaling, whereby RBCs control the bioavailability of vasoactive factors that modulate vessel caliber in an O_2_-dependent manner (i.e., S-nitrosothiols and ATP) (Ross et al., 1962; Frandsenn et al., 2001; Doctor and Stamler, 2011), and biophysical effects, via the influence of RBCs on blood rheology (determined by RBC deformability), aggregation (with each other), and adhesion (to endothelium). Together, these functions place RBCs at the center of O_2_ homeostasis regulation.

The highly infectious coronavirus 2019 disease (COVID-19) caused by severe acute respiratory coronavirus 2 (SARS-CoV-2) (Zhu et al., 2020) is characterized by impaired O_2_ delivery homeostasis. The COVID-19 virus S1 spike protein is postulated to interact with RBCs via RBC CD147 (Wang et al., 2020), evidenced by the detection of RBC surface viral spike protein and complement activation products (Lam et al., 2020; Bouchla et al., 2021). Additionally, COVID-19 spike protein glycans may also bind to glycoconjugates on the surface of red blood cells (Boschi et al., 2022). These interactions, in addition to the acute inflammation, which is a feature of COVID-19 (Wong, 2021) [and already known to affect RBC rheological properties (Pretorius, 2018)], likely affect RBC surface chemistry and rheology, which are proposed to result in intravascular thrombosis (Weisel and Litvinov, 2019), associated lung injury (Lam et al., 2021), and hypoxemia (Montenegro et al., 2021). Additionally, COVID-19 subjects often present with anemia, the severity of which appears greatest in those most critically ill (Chen et al., 2021), although it is not clear if this effect arises from increased RBC hemolysis and/or clearance, reduced hematopoiesis, or a combination of these factors. Unless compensated through homeostatic adaptation of blood flow and/or Hb–O_2_ affinity, reduced O_2_-carrying capacity diminishes O_2_ capacitance (i.e., the amount of O_2_ released across any given arteriovenous pO_2_ difference) (Mairbaurl and Weber, 2012), thereby loading and placing undue strain on the cardiovascular system to compensate for reduced O_2_ delivered per mL blood, which requires work to increase cardiac output in the setting of reduced capacity for myocardial O_2_ delivery, potentially contributing to disease pathogenesis.

Despite the fundamental RBC role in O_2_ homeostasis and the known impact of COVID-19 on O_2_ delivery homeostasis, the effect of COVID-19 on RBC properties and physiology is not well described nor quantified. The aim of this study was to determine whether RBCs from hospitalized COVID-19 subjects demonstrated altered features (or impaired compensation) relevant to O_2_ homeostasis, i.e., hematological parameters, altered O_2_ transport characteristics (i.e., Hb–O_2_ affinity/cooperativity), impaired rheology, and/or altered release of vasoactive compounds, i.e., a diminished hypoxic vasodilatory reflex. Additionally, given the ABO blood-type dependence on susceptibility to COVID-19 infection and spike protein–RBC interaction (Barnkob et al., 2020; Severe Covid et al., 2020; Wu et al., 2023), we assessed whether changes observed in features relevant to O_2_ homeostasis were associated with blood type.

## Materials and methods

### Subjects

This study was approved by the UMB Human Research Ethics Committee, and written informed consent was obtained from all participants in accordance with the Declaration of Helsinki. We studied 18 COVID-19 subjects who were hospitalized at the University of Maryland Medical Center (Baltimore, United States) between September 2020 and January 2022 and 20 non-hospitalized healthy control individuals. SARS-CoV-2 infection was confirmed in all subjects by polymerase chain reaction (PCR) performed on material collected using the nasopharyngeal swab. Medical records were reviewed to collect demographic and general clinical data.

### Blood sampling and processing

Venous blood was drawn into EDTA or heparin vacutainers and immediately placed on ice until analysis, which was performed within 24 h of collection. For complete blood count (CBC), oxygen dissociation curve (ODC—Hemox), and ektacytometry (LORRCA) analyses, whole blood was used, so no further processing was performed. RBC counts (CBC HORIBA) were adjusted for each assay platform (following the addition of whole blood to assay buffer) according to assay specifications. For hypoxic vasodilation (HVD), RBCs were separated from whole blood and washed three times (2,000 g, 10 min, 4°C, PBS) prior to analysis. Blood samples were equilibrated at the appropriate assay temperature prior to measurement.

### Materials and reagents

Unless stated, reagents were purchased from Sigma-Aldrich Inc. (St. Louis, United States).

### Hematological parameters and oximetry

Standard hematological parameters were measured in the clinic or research laboratory using conventional complete blood count analyzers (HORIBA, Kisshoin, Japan).

### Hemorheological parameters

RBC osmotic fragility, deformability, and aggregation were measured through ektacytometry using LORRCA (RR Mechatronics, Hoorn, Netherlands). This assay quantifies cell deformability as an elongation index [i.e., a ratio in the difference between the major and minor axes in cellular diffraction patterns over their sum (Lazarova et al., 2017; Gutierrez et al., 2021)], whilst cells are under constant shear. Three different measurements were performed: 1) osmoscan, yielding a set of elongation indices measured at 37°C and a single shear (30 Pa), across a wide osmotic gradient (∼80–700 mOsm); 2) deformability scan, yielding a set of elongation indices at 37°C and a fixed physiologic osmolality, across a shear range (0.3–30 Pascal); and 3) aggregation scan, i.e., a syllectogram, a time-dependent intensity plot of backscattered light generated when RBCs, initially subjected to shear (to fully induce disaggregation), are allowed to return to their original randomly oriented biconcave shape, lose alignment, and aggregate (as the shear is stopped) (Dobbe et al., 2003). Measurement outputs of aggregation included aggregation index (see Figure 4A) calculated as area A (the area within the rectangle above the syllectogram curve) divided by area A plus area B (the area within the rectangle below the syllectogram curve) multiplied by 100, the amplitude of aggregation, and t_1/2_. Whole blood was used (aggregation) or added and thoroughly mixed in an iso-osmolar polyvinylpyrrolidone (EIon ISO solution for osmoscan and deformability measurements—5 mL; RR Mechatronics, Hoorn, Netherlands; mean viscosity ∼29.8 mPa*s, osmolality ∼285 mOsm, pH ∼7.4), allowing normalization of the RBC count for each specific assay platform (as outlined by the manufacturer).

### O_2_ transport parameters

O_2_–hemoglobin dissociation curves were composed from the simultaneous direct *in vitro* measurement of O_2_ partial pressure (pO_2_) and hemoglobin O_2_ saturation (HbSO_2_) during controlled hemoglobin deoxygenation (O_2_ unloading) and re-oxygenation (O_2_ loading) (Guarnone et al., 1995) (Hemox-Analyzer, TSC Scientific Corporation, New Hope, PA, United States). Whole blood (25 μL was diluted in 3 mL 50 mM BIS-Tris, and 100 mM NaCl buffered to either pH 7.2, 7.4, or 7.6) (Benesch et al., 1969) with the addition of bovine serum albumin (BSA) (12 μL) and an antifoaming agent (6 μL), both supplied by the manufacturer. Samples were equilibrated (37°C) while bubbled with air and then subsequently deoxygenated, with exposure to N_2_. The Hb p50 value (pO_2_ at which HbSO_2_ is 50%) was extrapolated from the plotted relationship of the above two variables (oxy-hemoglobin desaturation curve; ODC). In addition, the Hill coefficient was calculated (TCS Hemox Data Acquisition System, TCS Scientific Corp, New Hope, PA, United States), providing an index of cooperativity, and the Bohr plot was determined (Guarnone et al., 1995). Additional analyses were performed from ODC data, including the calculation of blood O_2_ content from the O_2_ unloading arm of the Hemox ODC curves; SO_2_ was converted to blood O_2_ content assuming that 1 g Hb binds to 1.34 mL O_2_ and multiplying this by the subjects [Hb]. Blood O_2_ capacitance was also calculated from the ODC, using the calculation of Mairbaurl and Weber (2012).

### RBC vasoactivity (hypoxic vasodilation)

Male New Zealand white rabbits (1.8–2 Kg) were euthanized by intravenous injection of sodium pentobarbital. The aorta was harvested, and endothelium-intact rings were prepared for isometric tension recordings by mounting on a Radnoti vascular ring array (Harvard Apparatus, Holliston, MA, United States): 2 g resting tension, 37°C, and Krebs (NaCl 118 mM, KCl 4.8 mM, KH_2_PO_4_ 1.2 mM, MgSO_4_ 1.2 mM, NaHCO_3_ 24 mM, glucose 11.0 mM, CaCl_2_ 2.5 mM, and disodium EDTA 0.03 mM); bath pO_2_ was controlled by bubbling appropriate gas mixtures, as described (Pinder et al., 2009). Isometric tension was recorded continuously by transducers linked to a PowerLab 8SP/octal bridge (AD Instruments, Colorado Springs, CO, United States) connected to a PC running LabChart 7 (AD Instruments, Colorado Springs, CO, United States). Rings were pre-conditioned at 95% O_2_, 5% CO_2_, with 10^−6^ mol/L phenylephrine (PE) and 10^−5^ mol/L acetylcholine (ACh); then, under hypoxia (95% N_2_, 5% CO_2_; ∼1% O_2_), PE (5 × 10^−6^ mol/L) was used to increase baseline tension, before 30 μL of pelleted RBCs were injected into each bath, and the data were analyzed, as outlined previously (James et al., 2004). In brief, endothelium-intact rings were identified as those producing an ACh relaxation response during preconditioning that was >60% of the maximal induced tension (by PE); rings that did not produce this amount of relaxation were excluded. For each experiment, eight ring preparations were run in parallel, *n* = 1 represents data averaged from all endothelium-intact rings (possibly 8 in total) that were treated identically; the % relaxation was calculated as the RBC-induced decrease in tension as a percentage of the preceding baseline plateau tension (both under the above hypoxic bath conditions) (James et al., 2004).

### Statistical analyses

Results are presented as the mean ± standard deviation (SD) (or SEM where indicated). Column statistics were performed, and a normality/lognormality test (Shapiro–Wilk test) was undertaken to confirm normal data distribution. Data were plotted as box and whisker plots with each data point shown (box extending from the 25th to 75th percentile) and the whiskers representing minimum and maximum points. For parametric data, group comparisons of means were analyzed using the *t*-test (Student’s). For non-parametric data, group comparisons of mean ranks were analyzed using Mann–Whitney *U*-test (Prism, GraphPad Inc.; La Jolla, CA). Curves (Figures 3E, 5F, 8E) were compared via two-way ANOVA (mixed-effects analysis), and comparison of means at each shear (3E, 8E) or each lung-to-tissue O_2_ flux (5F) between groups were performed. Pearson’s product moment correlation coefficient was computed to assess the relationship between mean corpuscular hemoglobin concentration (MCHC)/mean corpuscular volume (MCV) and RBC deformability (Figure 3H). A *p*-value <0.05 was considered significant. For original data please contact Allan Doctor at ADoctor@som.umaryland.edu.

## Results

### Patient demographics and clinical presentation

Healthy age/sex-matched controls were compared with COVID-19 subjects (sex distribution; chi-squared test (*X* ^*2*^ 1, N = 38) = 0.095, *p* = 0.758; mean age; COVID-19: 52.6 ± 12.5 years vs healthy control 46.1 ± 9.4 years, *p* = 0.074) (Table 1). As is typical of hospitalized subjects in intensive care, COVID-19 subjects presented with multiple comorbidities (Table 1). As a gauge of COVID-19 severity, we report the mean Simplified Acute Physiology Scores (SAPS II) (25.6 ± 11.6 AU), in addition to the fact that 3 of the 18 COVID-19 subjects (17%) died during their hospitalization stay (Table 1).

**TABLE 1:** Healthy control and COVID-19 subject demographics.

		Healthy			COVID-19		
		Male	Female	Total	Male	Female	Total
Demographics	N	9	11	20	9	9	18
	Age (mean)	45.4 ± 9.8	46.5 ± 9.6	46.1 ± 9.4	50.8 ± 15.4	54.4 ± 9.2	52.6 ± 12.5
	Age [median (25th and 75th percentile)]	44 [41; 52]	54 [35; 54]	44 [39; 54]	55 [35; 64]	58 [45; 62]	58 [42; 62]
Comorbidities	Obesity	—	—	—	5 (56%)	7 (78%)	12 (67%)
	Hypertension	—	—	—	6 (67%)	6 (67%)	12 (67%)
	Diabetes	—	—	—	1 (11%)	8 (89%)	9 (50%)
	Heart failure	—	—	—	3 (33%)	3 (33%)	6 (33%)
	Hyperlipidemia	—	—	—	0 (0%)	5 (56%)	5 (28%)
	Chronic kidney disease (CKD)	—	—	—	4 (44%)	1 (11%)	5 (28%)
	Sample collection (days from symptom onset)	—	—	—	13.4 ± 10.3	11.3 ± 6.4	12 (67%)
Severity	SAPS II score	—	—	—	27.3 ± 13.1	23.0 ± 9.5	25.6 ± 11.6
Outcome	Death (%)	0	0	0	2 (22%)	1 (11%)	3 (17%)

### Clinical hematologic parameters—healthy controls vs COVID-19 subjects

**FIGURE 1:**
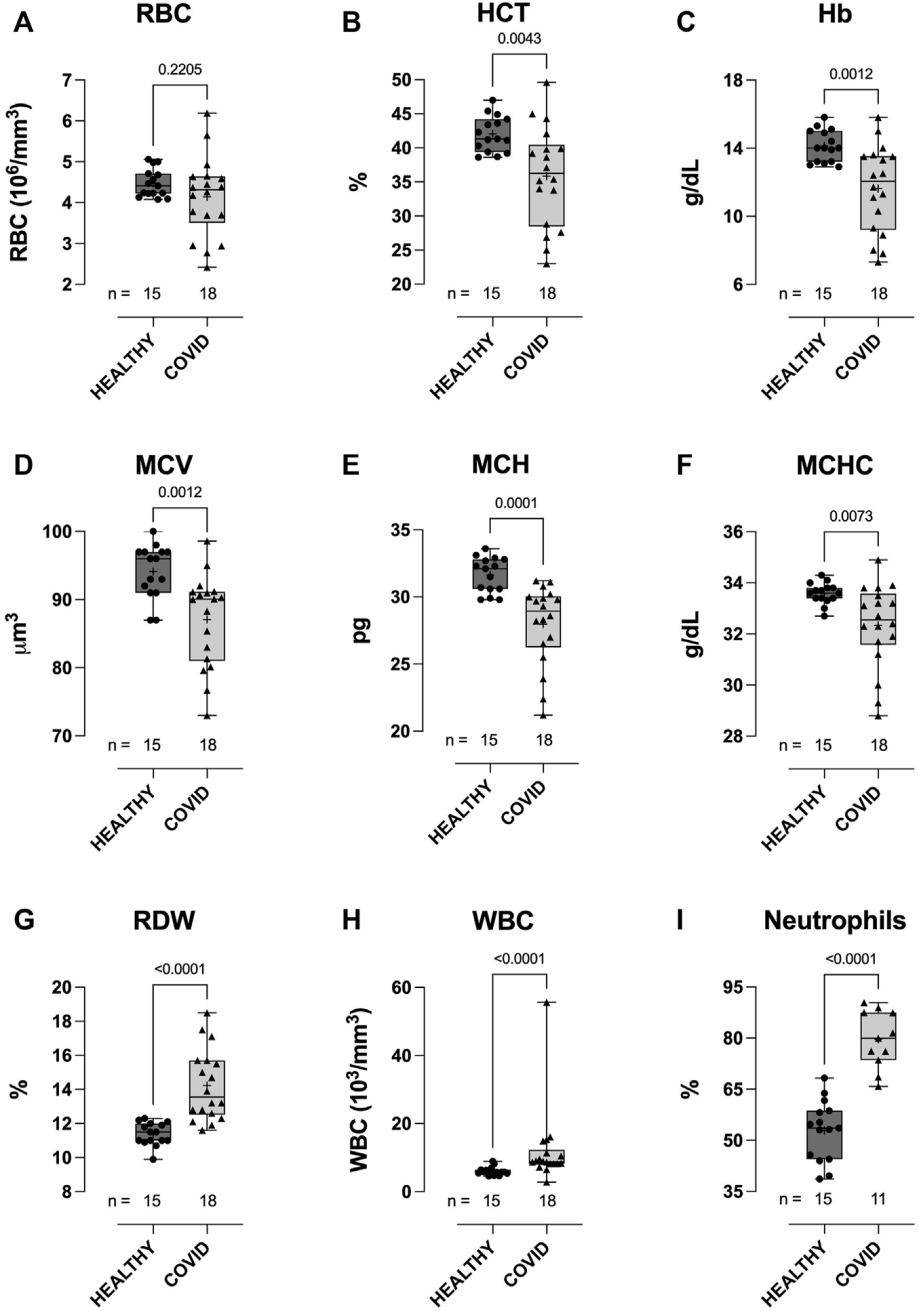
Clinical hematological parameters of healthy control and hospitalized COVID-19 subjects. Compared to healthy controls, blood samples from COVID-19 subjects demonstrated higher white blood cell (WBC) counts **(H)**, with a higher percentage of neutrophils **(I)**. No difference between groups was observed in red blood cell (RBC) counts **(A)**. COVID-19 subjects also demonstrated lower hematocrits (HCT) **(B)**, hemoglobin concentration [Hb] **(C)**, mean corpuscular volume (MCV) **(D)**, mean corpuscular hemoglobin **(E)**, mean corpuscular hemoglobin concentration (MCHC) **(F)**, and higher red cell distribution width (RDW) **(G)**. Data are presented as the box and whisker plot. The median is indicated by a solid line, and the mean is represented by +. Notably, a subset of controls (*n* = 5) did not have CBC measures performed. In addition, neutrophil counts were not available for some of the COVID-19 subjects.

The WBC count was higher in hospitalized COVID-19 subjects than in healthy controls (12.1 ± 11.32 vs 6.1 ± 1.2: 103/mm^3^, *p* < 0.0001; Figure 1H), and WBC counts from the COVID-19 subjects comprised a significantly higher percentage of neutrophils than those from healthy controls (79.6 ± 8.4 vs 52.9 ± 8.8: %, *p* < 0.0001; Figure 1I). RBC counts did not differ between groups (Figure 1A). Compared to healthy controls, hematocrit (35.9 ± 7.38 vs 42.0 ± 2.62: %, *p* = 0.0043; Figure 1B), Hb concentration (11.6 ± 2.42 vs 14.1 ± 0.93: g/dL, *p* = 0.0012; Figure 1C), MCV (87.1 ± 6.75 vs 94.1 ± 3.94: μm^3^, *p* = 0.0012; Figure 1D), mean corpuscular hemoglobin (MCH; 28.0 ± 2.99 vs 31.7 ± 1.31: pg, *p* = 0.0001; Figure 1E), and MCHC (32.3 ± 1.64 vs 33.6 ± 0.41: g/dL, *p* = 0.0073; Figure 1F) were all lower in COVID-19 subjects, whilst RBC distribution width was higher (14.2 ± 2.08 vs 11.4 ± 0.67: %, *p* < 0.0001; Figure 1G).

### Hemorheological parameters—healthy controls vs COVID-19 subjects

**FIGURE 2:**
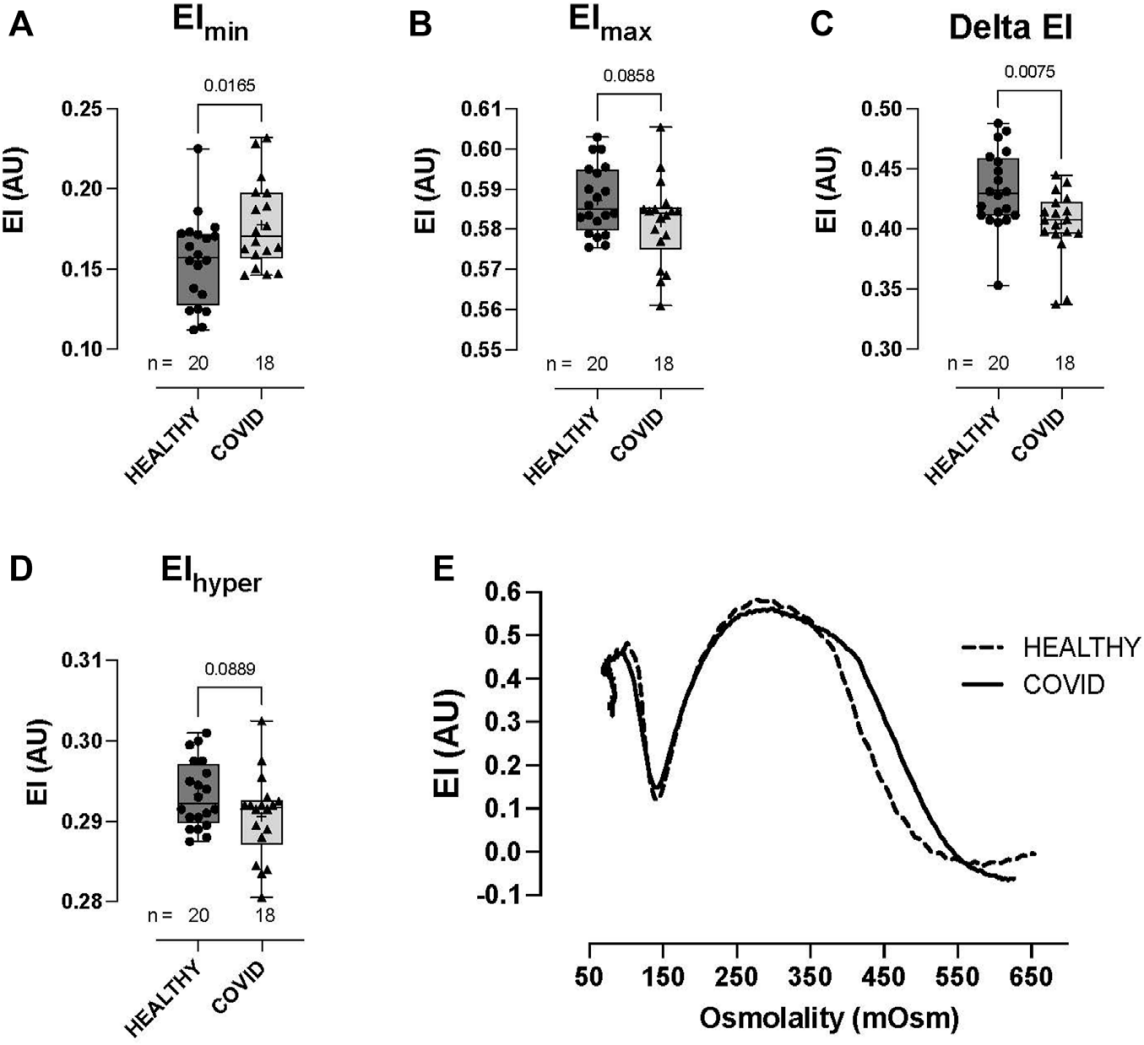
Shear-induced RBC osmotic fragility in healthy controls and hospitalized COVID-19 subjects measured using LORRCA. Compared to healthy control RBCs, COVID-19 RBCs demonstrated significant impairment in cell volume regulation, with higher elongation index minimum, EImin **(A)**, lower elongation index maximum, EImax **(B)**, lower delta elongation index **(C)**, and elongation index hyper **(D)**. No differences were observed in buffer osmolality at EImin (Omin), EImax (OEImax), the delta osmolality, or hyperosmolality (Ohyper). Representative traces from healthy controls and COVID-19 subjects **(E)**. Data are presented as the box and whisker plot. The median is indicated by a solid line, and the mean is represented by +.

RBCs from COVID-19 subjects demonstrated altered ability to control the cell volume across an osmotic gradient (∼100–500 mOsm) under shear (30 Pa). At low osmolality (∼140 mOsm—at EImin), COVID-19 RBCs showed higher EI than healthy control RBCs (0.177 ± 0.027 vs 0.155 ± 0.028, respectively: EImin, *p* = 0.0165; Figure 2A). This hypotonic osmolality coincides with the osmolality at which 50% of the cells would hemolyze in an osmotic fragility assay (Clark et al., 1983), suggesting a lower surface area-to-volume ratio of the COVID-19 cells compared to the healthy controls and/or a loss of cell volume regulation. At physiologic and hyper-osmolality, RBC deformability in the COVID-19 subjects trended lower than that in healthy controls, although this difference was not statistically significant (EImax *p* = 0.0858 and EIhyper *p* = 0.0889; Figures 2B, D). The dynamic range in deformability across the measured osmotic gradient (deltaEI) was significantly lower in RBCs from COVID-19 subjects than those from controls (0.404 ± 0.028 vs 0.433 ± 0.032, respectively: deltaEI, *p* = 0.0075; Figure 2C). Buffer osmolalities at EImin, EImax, deltaEI, and EIhyper were not significantly different between the two groups (Figure 2E—representative osmoscans).

**FIGURE 3:**
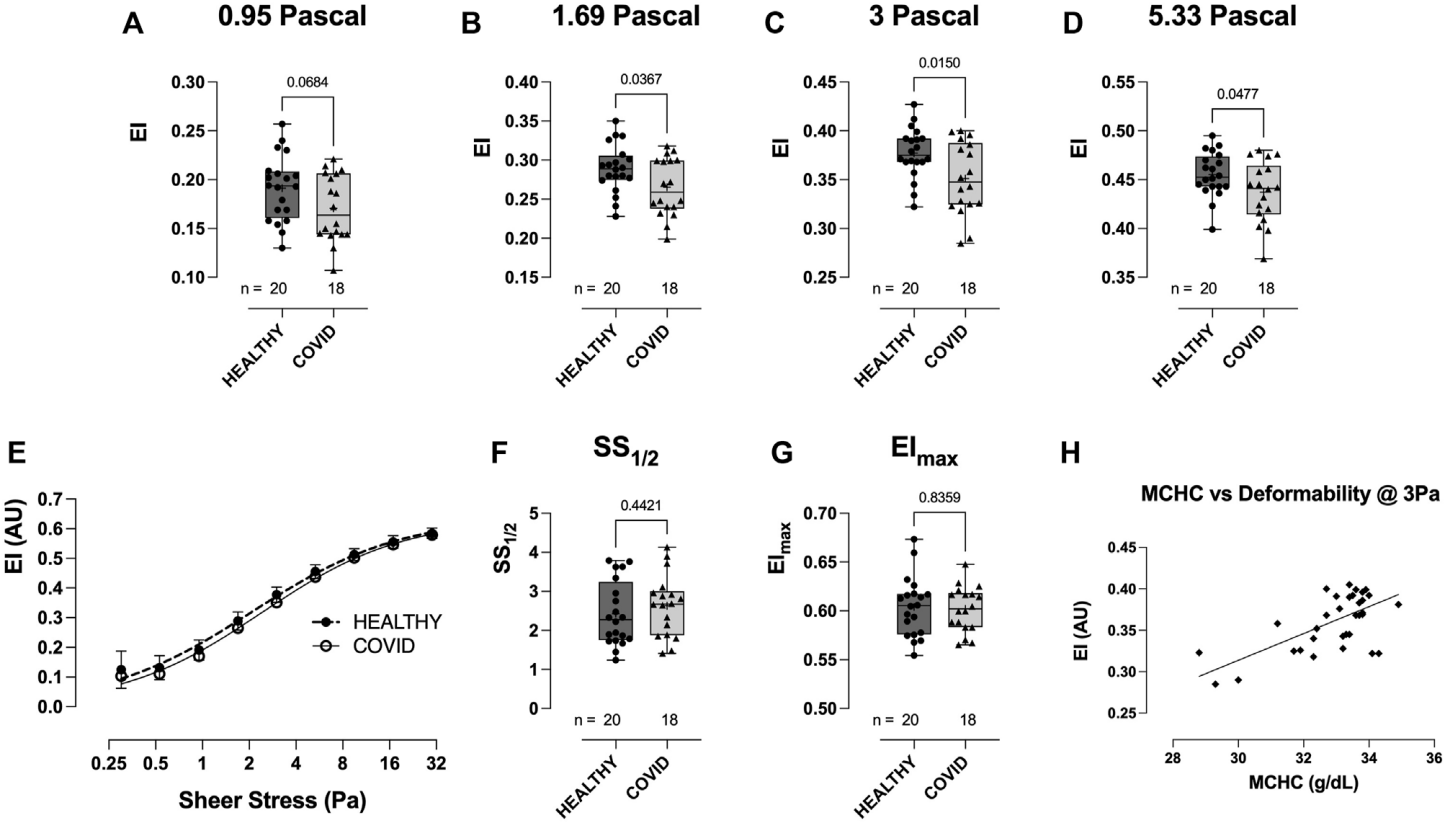
Shear-induced RBC deformability in healthy controls and hospitalized COVID-19 subjects, measured using LORRCA. Compared to healthy controls, RBC deformability was significantly reduced in hospitalized COVID-19 subjects across a range of shears: 0.95 **(A)**, 1.69 **(B)**, 3 **(C)**, and 5.33 Pa **(D)**. Deformability curve **(E)** and the calculated values from the deformability curves plotted, including SS1/2 **(F)** and EImax **(G)**. Correlation between MCHC and RBC deformability (at 3 Pa) plotted to show the relationship between these variables **(H)**. Data are presented as the box and whisker plot. The median is indicated by a solid line, and the mean is represented by +.

RBC deformability at fixed osmolality (∼285 mOsm) across a range of shears (0.3–30 Pascal; Pa) was significantly lower in the hospitalized COVID-19 subjects than in healthy controls (Figure 3). Importantly, this difference between groups was observed in the physiologic shear range (i.e., 1.69 Pa, *p* = 0.0367; 3 Pa, *p* = 0.0150; 5.33 Pa, *p* = 0.0477; Figures 3A–D). No difference was observed in the deformability curves between the healthy controls and COVID-19 subjects (Figure 3E), as confirmed in the analysis of SS1/2 (Figure 3F) and the calculated EImax (Figure 3G). Given that cell geometry dictates that MCHC (and MCV) are linked to the ability of RBCs to deform, we assessed the relationship between MCHC/MCV and deformability (at 3 Pa) and observed an expected significant correlation between these parameters (MCHC vs deformability = *r* (31) = 0.453, *p* < 0.0001; Figure 3H; MCV vs. deformability = *r* (31) = 0.17, *p* = 0.0172; data not shown).

**FIGURE 4:**
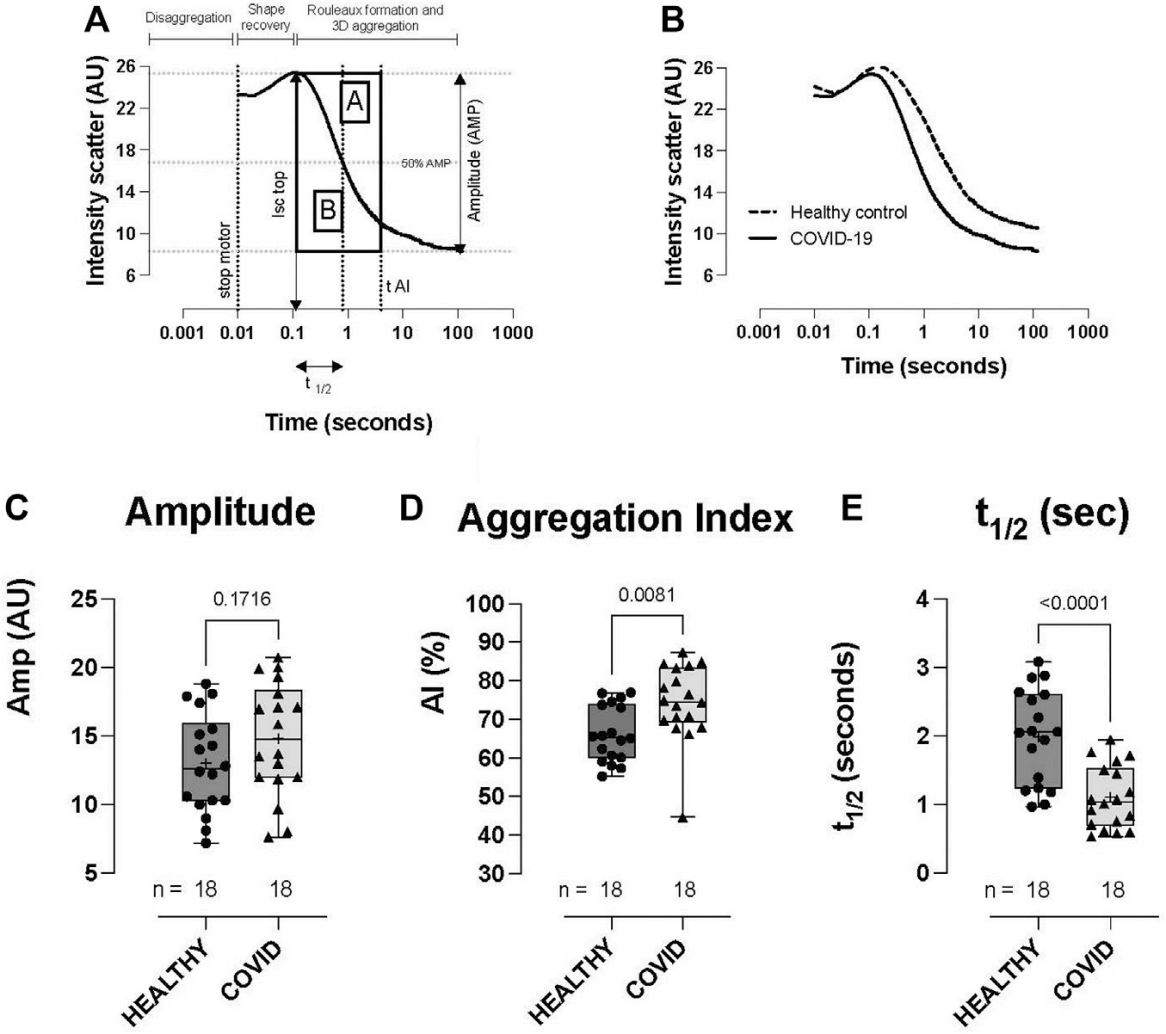
RBC aggregation (syllectogram) in healthy controls and hospitalized COVID-19 subjects, measured using LORRCA. Syllectogram of RBC aggregation plotted on a logarithmic timescale demonstrating the indices plotted **(A)**. Representative syllectogram traces from healthy controls and COVID-19 subjects **(B)**. No difference was observed in the total aggregation response between healthy controls and COVID-19 subjects (amplitude) **(C)**. Aggregation profile (aggregation index) **(D)** and the kinetics of aggregation (t1/2) **(E)** were significantly different between the groups, with COVID-19 subjects demonstrating higher AI% and a shorter time to reach half-maximal aggregation (t1/2). Data are presented as the box and whisker plot. The median is indicated by a solid line, and the mean is represented by +. Notably, two control subjects did not have aggregation measurements performed.

Whilst the extent of RBC aggregation (Amplitude, AMP; AU) was not significantly different between the healthy controls and COVID-19 subjects (Figures 4A–C), RBC aggregation kinetics were significantly accelerated in the hospitalized COVID-19 subjects compared to healthy controls, as defined by the aggregation index (74.3 ± 9.96 vs 66.2 ± 7.27: AI%, *p* = 0.0081; COVID-19 vs healthy control) and t1/2 (1.10 ± 0.457 vs 1.99 ± 0.69: t1/2 s, *p* < 0.0001; COVID-19 vs healthy control; Figures 4A, B, D, E).

### RBC O_2_ transport parameters—healthy controls vs COVID-19 subjects

**FIGURE 5:**
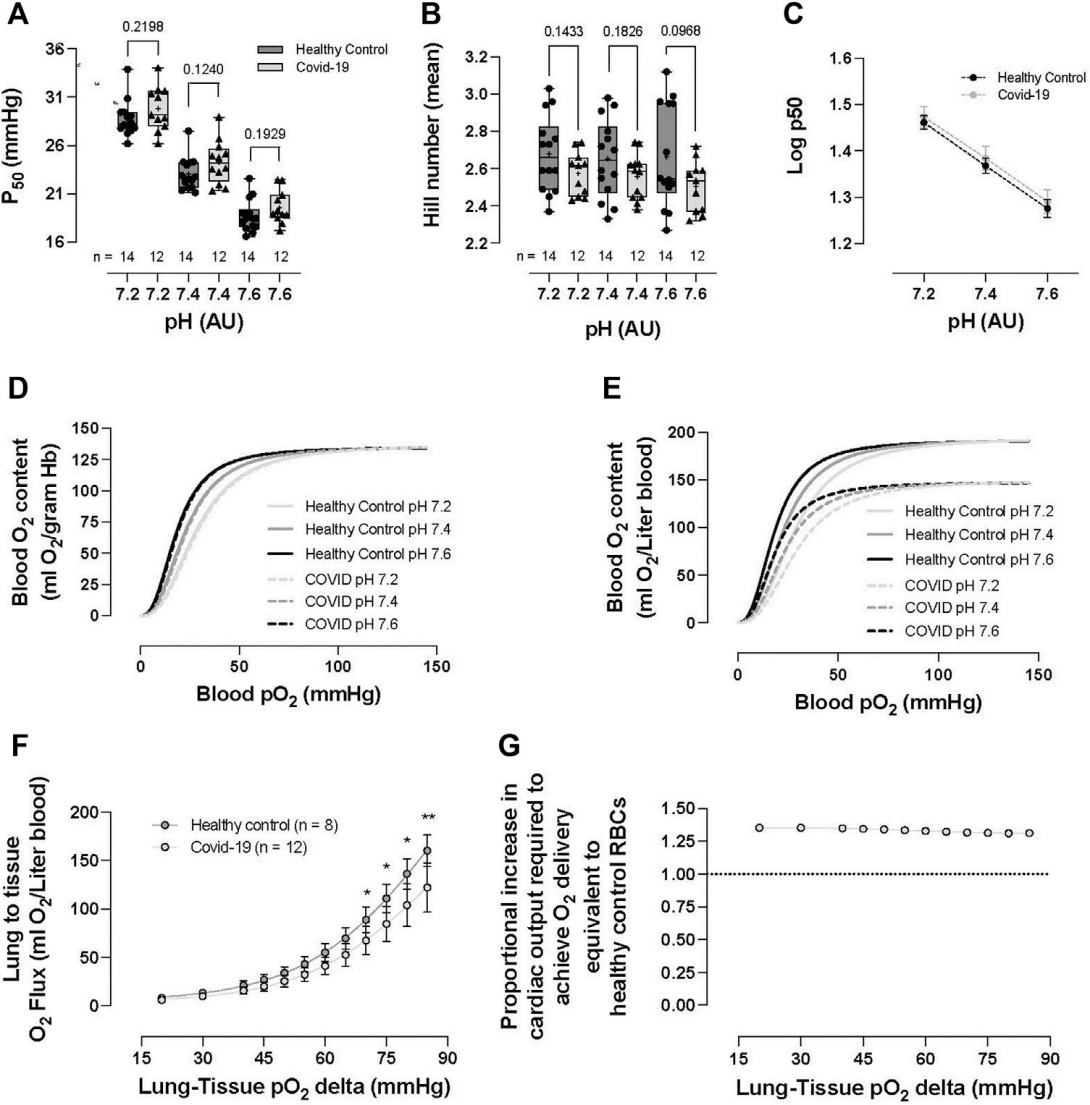
RBC O_2_ transport parameters of healthy controls and hospitalized COVID-19 subjects. No significant differences were observed between healthy controls or COVID-19 subjects in p50 (i.e., partial pressure of O_2_ that hemoglobin within RBCs 50% saturated with O_2_) **(A)**, Hb cooperativity as determined by the Hill number (mean) **(B)**, or Bohr effect **(C)**. No significant differences were observed in blood O_2_ content per gram Hb (calculated from Hemox ODCs) between healthy controls and COVID-19 RBCs **(D)**. Impact of anemia on O_2_ delivery evaluated via the calculation of O_2_ content per liter of blood (from ODC and CBC analyses). COVID-19 subjects demonstrated significantly reduced blood O_2_ content (ml O_2_/L) **(E)**. The reduction in blood O_2_ content was explored following the calculation of O_2_ capacitance (quantifying the amount of O_2_ unloaded for a given A-V pO_2_ difference). COVID-19 subjects presented with significantly reduced O_2_ capacitance, calculated from the Hb–O_2_ saturation at 100 mmHg, taken from the O_2_ loading curve measured at pH 7.4 (reflecting O_2_ uptake conditions in the lung) and the corresponding Hb–O_2_ saturations at various pO_2_ values taken from the O_2_ unloading curve measured at pH 7.2 (reflecting O_2_ unloading from RBCs at the tissue level) (Mairbaurl and Weber, 2012) **(F)**. To overcome the O_2_ deficit, the proportional increase in cardiac output necessary to achieve O_2_ delivery equivalence to healthy control RBCs was calculated (need in COVID-19 subjects was ∼1.3–1.4 times that of healthy control individuals) **(G)**. Data are presented as the box and whisker plots. The median is indicated by a solid line, and the mean is represented by + **(A,B)**. Data plotted as the XY plot ± SD **(C)**, **(G)** plotted without error bars, **(D–E)** plotted as mean ± SD, **(F)** plotted as the proportion between healthy controls and COVID-19 subjects. Notably, Hemox analysis was not performed on all subjects, and some subjects did not undergo CBC analysis, allowing the calculation of blood O_2_ content or O_2_ capacitance.

No significant difference was observed in RBC–O_2_ affinity (i.e., p50) between the healthy control and COVID-19 subjects across three pH measurements (pH 7.2: 28.77 ± 1.87 vs 29.83 ± 2.31, *p* = 0.2198; pH 7.4: 23.06 ± 1.69 vs 24.3 ± 2.25, *p* = 0.124; and pH 7.6: 18.69 ± 1.67 vs 19.59 ± 1.72, *p* = 0.1929, healthy control vs. COVID-19, respectively; Figure 5A). Hb–O_2_ cooperativity was not significantly different between the healthy controls or COVID-19 subjects (pH 7.2: 2.68 ± 0.20 vs 2.57 ± 0.11, *p* = 0.1433; pH 7.4: 2.65 ± 0.21 vs 2.56 ± 0.12, *p* = 0.1826; and pH 7.6: 2.66 ± 0.28 vs 2.51 ± 0.14, *p* = 0.0968, healthy control vs COVID-19, respectively; Figure 5B). The Bohr effect (i.e., the shift in the ODC in response to pH change), tested by running ODCs at three fixed pH levels, was not different between the healthy control and COVID-19 groups (Figure 5C). We calculated the mean total blood O_2_ content as a function of blood pO_2_ for each pH, per gram Hb (Figure 5D) and per Liter blood (Figure 5E) after calculating blood O_2_ content from acquired data [i.e., for per gram Hb calculation, SO_2_ was converted to blood O_2_ content given that 1 g hemoglobin binds to 1.34 mL O_2_, for per Liter blood calculation, SO_2_ was converted to blood O_2_ content per gram Hb and then multiplied by each subject’s [Hb]; (Figure 5D); this analysis demonstrates the significant reduction in blood O_2_ content between the COVID-19 subjects and healthy controls for similar O_2_ tensions. Next, we calculated O_2_ capacitance [absolute amount of O_2_ released by RBCs upon transit across a given physiologic O_2_ gradient (lung → tissue)] (Mairbaurl and Weber, 2012) from the Hb–O_2_ saturation at 100 mmHg, using an O_2_ loading curve at pH 7.4 (representing RBC O_2_ content in pulmonary veins) and the Hb–O_2_ saturation at various pO_2_ values on the O_2_ dissociation curve measured at pH 7.2 (representing RBC O_2_ content in perfused tissue). These data, which integrate both O_2_-carrying capacity and Hb–O_2_ affinity, quantify the amount of O_2_ unloaded across the physiologic range of arterio-venous (A-V) pO_2_ differences encountered during circulatory transit. We observed a significant, progressive reduction in O_2_ capacitance in COVID-19 subjects for A-V O_2_ gradients >70 mmHg (i.e., equivalent to tissue pO_2_ < 30 mmHg, *p* < 0.05; Figure 5F), with RBCs from COVID-19 subjects demonstrating capacity for only ∼70–75% of the calculated lung-to-tissue O_2_ flux (mL) across the physiologic O_2_ gradient compared to that for healthy controls (Figure 5F).

### RBC vasoactivity (HVD response)—healthy controls vs COVID-19 subjects

No difference was observed in the hypoxic vasodilatory response of RBCs from healthy controls vs COVID-19 subjects, normalized to the maximal PE constriction of the respective vascular rings (8.67% ± 1.9% vs 9.4% ± 2.3%, *p* = 0.413, control vs COVID-19, respectively—data not shown).

### RBC properties COVID-19 blood type

Analysis was performed to assess whether blood type influenced RBC properties in the hospitalized COVID-19 subjects. Patient numbers and demographics between the two majority blood-type groups in our cohort (i.e., A and O; sex distribution, age, and sample collection time from disease onset) were not significantly different (*p* > .05–Table 2).

**TABLE 2:** COVID-19 subject demographics by blood type.

		Blood type A	Blood type O
Demographics	Age (mean)	52.6 ± 13.2	57.2 ± 8.6
	Age [median (25th and 75th percentile)]	59 [47; 61]	58 [52; 64]
	Subject number	7	6
	Sex (M/F)	3/4	3/3
	Obesity	5 (71%)	3 (50%)
	Hypertension	4 (57%)	5 (83%)
	Diabetes	5 (71%)	3 (50%)
	Heart failure	4 (57%)	2 (33%)
	Hyperlipidemia	3 (43%)	1 (17%)
	Chronic kidney disease (CKD)	2 (29%)	2 (33%)
	Sample collection (days from symptom onset)	10.3 ± 7.8	14.7 ± 11.7
Severity	SAPS II score	26.6 ± 8.8	30.0 ± 14.0
Outcome	Death (%)	2 (29%)	1 (17%)

### Clinical hematologic parameters—COVID-19 blood type

**FIGURE 6:**
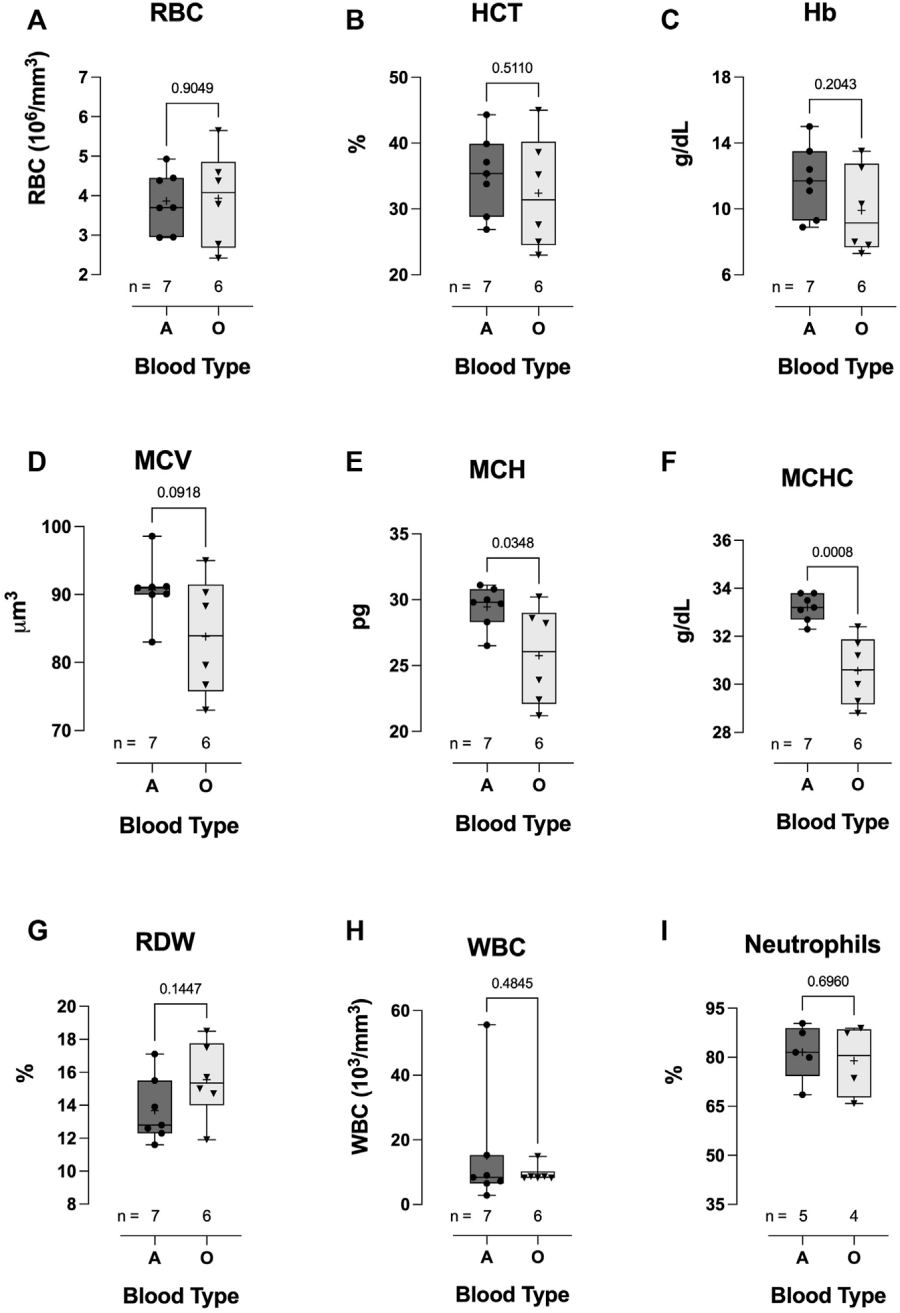
Effect of blood-types A and O on COVID-19 clinical hematological parameters. No significant differences were observed in WBC count **(H)**, neutrophil % **(I)**, RBC count **(A)**, HCT **(B)**, Hb concentration **(C)**, MCV **(D)**, or RDW **(G)**. However, MCH **(E)** and MCHC **(F)** were significantly lower in blood-type O COVID-19 subjects than in blood-type A patients. Data are presented as the box and whiskers plot. The median is indicated by a solid line, and the mean is represented by +.

Hospitalized COVID-19 subjects with blood-type A or O showed similar hematological values (no difference between groups in RBC count, HCT, Hb concentration, MCV, RDW, WBC count, or neutrophil %) (Figures 6A–D, G–I). However, MCH (25.8 ± 3.72 vs 29.5 ± 1.58, *p* = 0.0348; Figure 6E) and MCHC were significantly lower in the blood-type O group than in the blood-type A group (33.2 ± 0.56 vs 30.6 ± 1.42, *p* = 0.0008; Figure 6F).

### Hemorheological parameters—COVID-19 blood type

**FIGURE 7:**
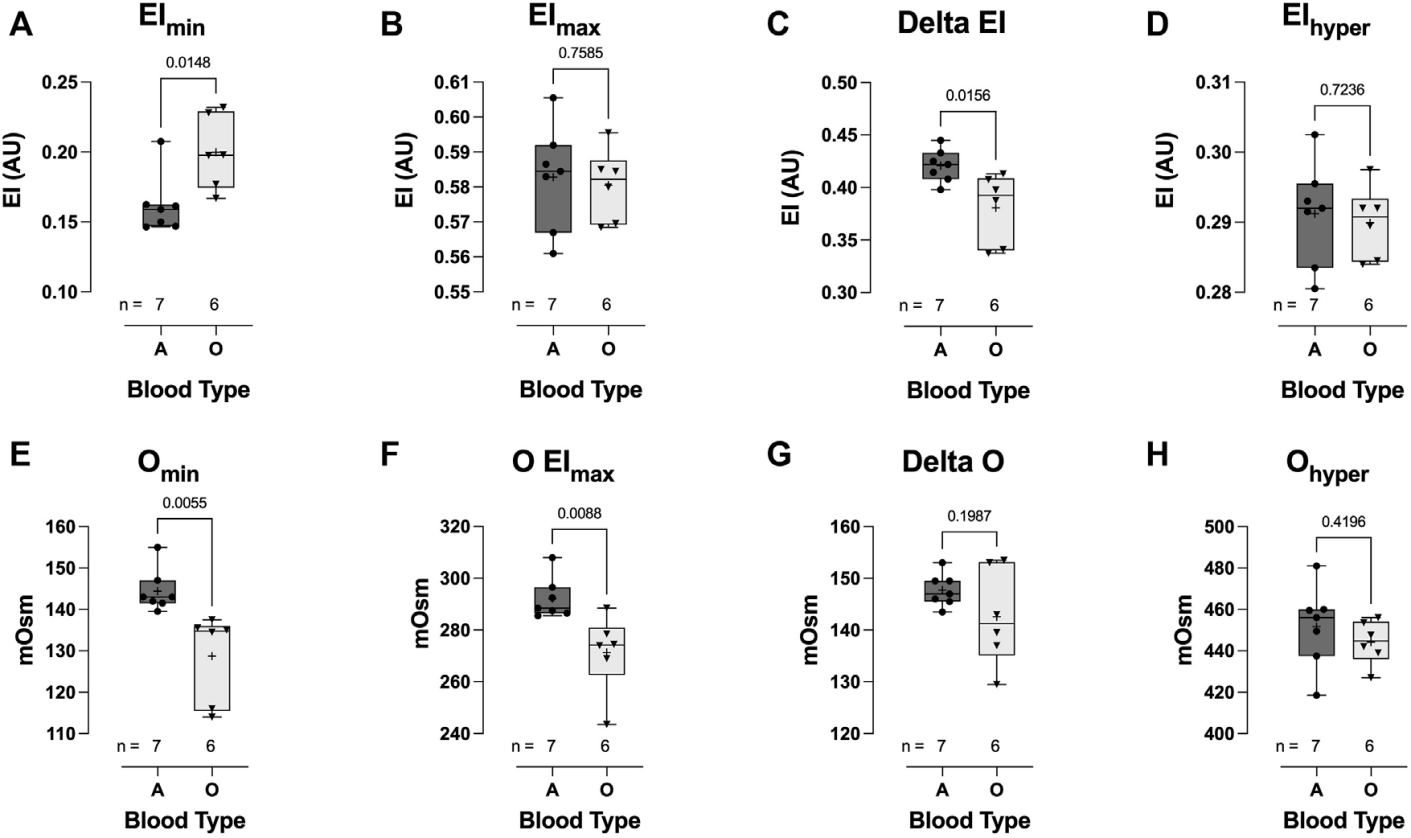
Effect of blood-types A and O on COVID-19 shear-induced RBC osmotic fragility. RBCs from COVID-19 subjects with blood-type O demonstrated significant impairment in cell volume regulation, with higher elongation index minimum (EImin) **(A)**, compared to those with blood-type A. No difference between blood types was observed in the elongation index maximum (EImax) **(B)**. Consequently, COVID-19 subjects with blood-type O demonstrated a significantly reduced delta elongation index **(C)** compared with subjects with blood-type A. No difference between blood types was observed in elongation index hyper **(D)**. The buffer osmolality at EImin (Omin) **(E)** and EImax (OEImax) **(F)** was significantly lower in blood-type O than in blood-type A subjects. However, the delta osmolality **(G)** and hyperosmolality (Ohyper) **(H)** were not different between blood types. Data are presented as the box and whiskers plot. The median is indicated by a solid line, and the mean is represented by +.

Hospitalized COVID-19 subjects with blood type O demonstrated significant impairment in the ability to regulate cell volume during osmotic stress. At low osmolality (∼140 mOsm), RBC deformability in the blood-type O COVID-19 patient group was significantly higher than that in the blood-type A group (0.200 ± 0.026 vs 0.162 ± 0.021, respectively: EImin *p* = 0.0148; Figure 7A). Once again, this hypotonic osmolality coincides with the osmolality at which 50% of the cells would hemolyze in an osmotic fragility assay (Clark et al., 1983), suggesting a lower surface area-to-volume ratio of the blood type-A COVID-19 cells compared to the blood-type O and/or a loss of cell volume regulation. At physiologic and hyperosmolality, RBC deformability was not different between the two groups (Figures 7B, D, respectively). The dynamic range for deformability across the osmotic gradient (deltaEI) was significantly lower in the blood-type O COVID-19 group than in the blood-type A group (0.381 ± 0.033 vs 0.421 ± 0.016, respectively: deltaEI *p* = 0.0156; Figure 7C). Furthermore, buffer osmolalities at EImin (i.e., Omin) and EImax (i.e., OEImax) were significantly lower in the blood-type O COVID-19 group than in the blood-type A group (Figures 7E, F, respectively) but not the DeltaO or Ohyper (Figures 7G, H, respectively).

**FIGURE 8:**
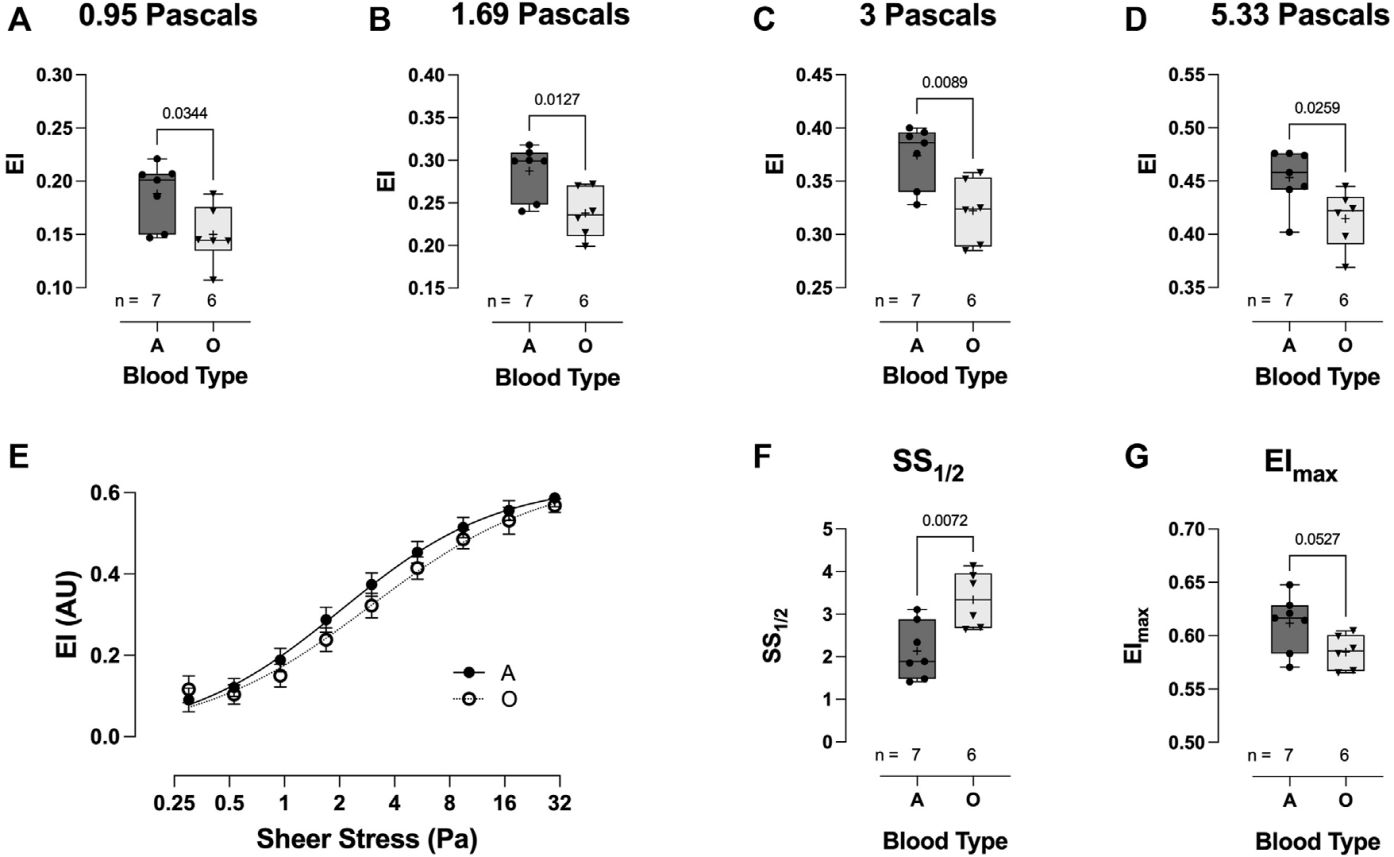
Effect of blood-types A and O on COVID-19 shear-induced RBC deformability. RBCs from COVID-19 subjects with blood-type A demonstrated significantly greater deformability at 0.95 **(A)**, 1.69 **(B)**, 3 **(C)**, and 5.33 Pa **(D)** than those from individuals with blood-type O. No differences were observed in the plotted deformability curves **(E)**; however, SS1/2 was significantly reduced in blood-type A than in blood-type O **(F)**, whilst calculated EImax was higher **(G)**. Data are presented as the box and whiskers plot. The median is indicated by a solid line, and the mean is represented by +.

Across a range of physiological shear stress (from 0.95 to 5.33 Pa), RBC deformability in the blood-type O hospitalized COVID-19 patient group was significantly lower than that in the blood-type A group (Figures 8A–E). Whilst no significant difference was observed in the deformability curves between blood-types O and A (Figure 8E), blood-type A subjects demonstrated a significantly reduced SS1/2 (3.34 ± 0.65 vs 2.14 ± 0.66, respectively: *p* = 0.0072; Figure 8F) and higher calculated EImax (0.58 ± 0.02 vs 0.61 ± 0.03, respectively: *p* = 0.0527; Figure 8G).

**FIGURE 9:**
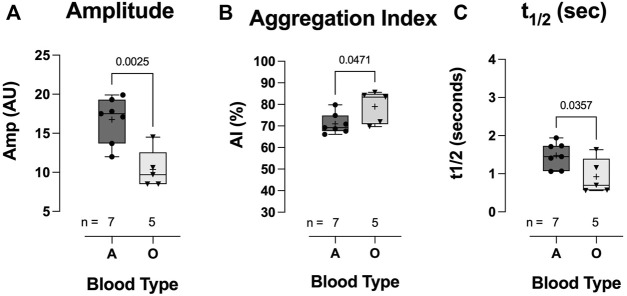
Effect of blood-types A and O on COVID-19 RBC aggregation. Individuals with blood-type A demonstrated significantly greater total aggregation (amplitude) **(A)** but a slower aggregation response, i.e., lower aggregation index **(B)** and longer t1/2 **(C)**. Data are presented as the box and whisker plot. The median is indicated by a solid line, and the mean is represented by +. Notably, one blood-type O individual did not have enough blood for aggregation measurement.

The total extent of RBC aggregation (AMP, AU) was significantly lower in the blood-type O COVID-19 group than in the blood-type A group (10.36 ± 2.48 vs 16.76 ± 2.89: AMP AU, *p* = 0.0025; Figure 9A). Additionally, the kinetics of RBC aggregation were significantly different between groups, with RBCs from hospitalized COVID-19 subjects with blood-type O aggregating faster than those with blood-type A (79.0 ± 7.5 vs 71.0 ± 4.7: aggregation index;, *p* = 0.0471; Figure 9B; 0.92 ± 0.46 vs 1.48 ± 0.34: t1/2 s, *p* = 0.0471; Figure 9C).

## Discussion

We quantified RBC features relevant to O_2_ delivery homeostasis in hospitalized COVID-19 subjects and observed 1) an altered hematological profile, with significantly elevated WBC counts, higher neutrophil levels, marked anemia (HCT and Hb), reduced RBC volume (MCV) and RBC [Hb] (MCH and MCHC), and increased RDW; 2) diminished O_2_-carrying capacity and O_2_ capacitance [integrated effect of lower (Hb) and lower O_2_ delivery per gram Hb across the physiologic O_2_ gradient]; and 3) impaired hemorheology, distinguished by (a) loss of cell volume regulation, (b) reduced RBC deformability, and (c) accelerated RBC aggregation kinetics, but 4) without change in the hypoxic vasodilatory reflex of RBCs. These COVID-19 disease RBC features were found to be exaggerated in hospitalized COVID-19 subjects with blood-type O compared to those with blood-type A.

Others have observed that hospitalized COVID-19 subjects present with anemia (i.e., RBC lack or diminished hemoglobin concentration) (Taneri et al., 2020; Bergamaschi et al., 2021) and hypoxemia (Montenegro et al., 2021). Blood O_2_-carrying capacity (i.e., blood O_2_ content) is essential to maintain O_2_ delivery homeostasis and is determined by both hemoglobin concentration and O_2_ affinity. Reduced blood O_2_-carrying capacity has obvious implications for tissue O_2_ delivery and potential O_2_ supply/demand gap, which is a risk factor for organ injury (Bergamaschi et al., 2021). Herein, we report reduced Hb concentration, hematocrit, MCV, MCH, and MCHC in our COVID-19 subjects, without a change in RBC count. The RBC count trended lower in the COVID-19 subjects than in healthy controls; however, the large variation in this measurement might explain the non-significant finding. These changes in RBC size, in addition to RBC [Hb], are indicative of microcytic hypochromic anemia, which has previously been observed in COVID-19 subjects (Elemam et al., 2022). However, we caution that some of these findings might partially be explained by comorbidity differences between the COVID-19 and healthy control populations.

Non-hemodynamic homeostatic adaptations counter the effect of anemia, increasing erythropoietin (EPO) (stimulating RBC production) and 2,3 DPG (right-shifting the ODC, aiding HbO_2_ offloading) and altering the intra-erythrocytic milieu (i.e., affecting pH). These adaptations can be quantified by measuring [Hb], reticulocyte count (RBC production), and the O_2_ dissociation curve (2,3 DPG and intra-RBC milieu changes), on which the p50 point is defined by the pO_2_ at which HbSO_2_ is 50%. In line with multiple other studies, we show no difference in the standard p50 between acute COVID-19 subjects and controls (Daniel et al., 2020; DeMartino et al., 2020; Renoux et al., 2021). However, we observed a non-significant trend toward higher p50 in the COVID-19 subjects across all three pH measurements. Considering that the reported effect of anemia typically elicits a p50 increase of ∼4 mmHg (∼26.7–∼30.7 mmHg; pH 7.4, 37°C), following a ∼50% reduction in [Hb] (resulting from reduced RBC production or loss of RBCs; ∼14.8 g/dL to ∼7.5 g/dL), with compensation provided by increased 2,3 DPG (∼12.7 μmol/gHb to ∼18.7 μmol/gHb) (Boning and Enciso, 1987); the variance in our data and lack of a statistically significant change in p50 might simply reflect the moderate degree of anemia in our COVID-19 population [(Hb) 11.6 ± 2.42 vs 14.1 ± 0.93: g/dL, *p* = 0.0012; Figure 1E]. Unfortunately, we did not measure 2,3 DPG in this study, which has been an issue with many COVID-19 studies, due to the lack of availability of test kits (Boning et al., 2021). Only one study to date reports on 2,3 DPG in COVID-19, showing a significant increase with moderate anemia compared to non-COVID subjects (measurement technique only reported arbitrary units) (Thomas et al., 2020).

O_2_ delivery homeostasis is not only a function of blood O_2_ content but also blood flow. In fact, the latter is the more important determinant, specifically because the dynamic range in O_2_ content is limited [varying linearly with (Hb) and percentage O_2_ saturation], whereas regional blood flow (a function of vessel radius to the fourth power) may be increased or decreased by several orders of magnitude. Consequently, it is the volume and distribution of blood flow that are modulated by physiologic reflexes that maintain dynamic coupling between O_2_ delivery and metabolic demand (Ross et al., 1962; Frandsenn et al., 2001). For the COVID-19 subjects to offset the reduction in blood O_2_-carrying capacity resulting from anemia (shown in Figure 5E), we would have expected a larger adaptive increase in p50 (Figure 5A) and a resulting greater difference between the healthy control and COVID-19 subjects in terms of blood O_2_ content per gram Hb (Figure 5D). Conversely, we observed diminished O_2_ capacitance (accounting for both the effect of anemia and lack of adaptation in HbO_2_ affinity, i.e., calculated as the amount of O_2_ unloaded for a given A-V pO_2_ difference from ODCs 16; Figure 5F). This finding quantifies the burden of increased cardiac output required to compensate for impaired blood O_2_ transport capacitance in COVID-19 subjects, which requires an increase of 130%–140% in cardiac output relative to that in healthy control individuals (Figure 5G, calculated from Figure 5F, i.e., the fold difference in O_2_ capacitance between COVID-19 subjects and healthy controls). This is particularly problematic since the demand for increased cardiac output burdens the myocardium with increased work despite concurrent diminished myocardial O_2_ delivery.

Numerous physical (i.e., deformability) and biochemical (i.e., factors which modulate vascular smooth muscle contractility) properties of RBCs play an essential role in determining microcirculatory flow (Lanotte et al., 2016) and as such play a significant role in maintaining O_2_ homeostasis. RBC biomechanical properties have been previously demonstrated to be altered in COVID-19 subjects (Kubankova et al., 2021; Grau et al., 2022), with RBCs exhibiting structural protein damage and membrane lipid remodeling (Thomas et al., 2020). We likewise demonstrate a significant reduction in RBC deformability across an isotonic shear stress gradient—LORRCA, RR Mechatronics 0.53–30 Pa (Figure 3). Deformability was also measured across an osmotic gradient (osmotic fragility—LORRCA Osmoscan), wherein we observed greater RBC deformability in RBCs from COVID-19 subjects than in those from healthy controls at low osmolality (i.e., higher EImin) in combination with a lower dynamic range in deformability across the osmotic gradient (i.e., lower deltaEI), suggestive of a lower surface area-to-volume ratio of the COVID-19 RBCs compared to the healthy controls, and/or a loss in the ability to regulate cell volume. A decrease in the surface area-to-volume ratio could be explained by increased blebbing and loss of damaged membrane from the COVID-19 RBCs, whilst loss of the ability to regulate cell volume might result from impaired RBC energetics, with reduced ATP for ion channel function, although the lack of change in p50 values between groups is suggestive of no critical change in ATP content. Furthermore, we also observed significant differences in RBC aggregation kinetics between the hospitalized COVID-19 subjects and controls, with COVID-19 enhancing aggregation, as shown by others (Nader et al., 2021; Renoux et al., 2021). In combination, these changes observed for RBCs from hospitalized COVID-19 subjects indicate rheological impairment that would be expected to significantly impair blood flow and contribute to dysregulation of O_2_ delivery homeostasis.

RBCs themselves also play an essential role in matching perfusion sufficiency to O_2_ demand via the release or scavenging of vasoactive signaling molecules (NO, NO+, and ATP). One such reflex is the regulation of hypoxic vasodilation (Ross et al., 1962; McMahon et al., 2000; Pawloski et al., 2001; McMahon et al., 2002; Buehler and Alayash, 2004; Singel and Stamler, 2005) through a series of O_2_-responsive, thiol-based transfers of NO groups to the endothelium (McMahon et al., 2002; Doctor et al., 2005; Palmer et al., 2007). We assessed the ability of RBCs to elicit the HVD response in the aortic tissue ring bath. Interestingly, we observed no difference in the HVD response between RBCs from hospitalized COVID-19 subjects and healthy controls. This was somewhat surprising, given the fact that others have shown higher levels of NO inside RBCs from COVID-19 hypoxemic subjects (Mortaz et al., 2020). Whilst the mechanism(s) causing the accumulation of intracellular NO in RBCs of COVID-19 subjects is still unclear, it appears that the higher NO content is not associated with an increase in vasoactive NO species release under hypoxic conditions, and thus, the physiologic relevance of this increased NO remains undefined.

Several recent studies have investigated the association between blood type and COVID-19 infection (Kim et al., 2021). Whilst it has been reported that blood-type A might be predisposed to increased susceptibility of infection with SARS-CoV-2 and type O and Rh-negative blood groups might be protective, no clear relationship appears to exist between blood type and COVID-19-related severity of illness or mortality. We looked to identify whether any relationship existed between blood type and RBC features related to maintenance of O_2_ homeostasis. In a limited subset of individuals, we compared RBCs of hospitalized COVID-19 subjects with blood type A or O. Somewhat surprisingly, we observed that the abnormal RBC properties associated with hospitalized COVID-19 subjects appeared exaggerated in blood-type O hospitalized COVID-19 subjects compared to blood type A. This is contrary to a reported putative protective role of blood-type O in terms of severity of illness and mortality.

We are aware of the limitations of our study. First, we acknowledge the small subject size, in addition to the fact that the hospitalized COVID-19 subjects 1) present with numerous comorbidities, which might also be expected to influence RBC physiology, in addition to 2) being given medications that might potentially have impacted the measurements herein (i.e., O_2_ affinity and NO metabolism). It is also likely that even though all subjects studied were hospitalized, which itself is an indication of disease severity, individuals studied had wide-ranging morbidity; thus, the variance in the data from COVID-19 subjects likely reflect wide-ranging subject morbidity. We also wish to highlight that acute inflammation itself is known to affect RBC rheological properties (Pretorius, 2018). Consequently, we cannot rule out that altered RBC features observed in hospitalized COVID-19 subjects may have resulted from the inflammatory response induced by COVID-19 and not directly from COVID-19 virus interaction with RBCs. Rheological studies have previously demonstrated blood sensitivity to anti-coagulant, short-term storage, and cold shock (Baskurt et al., 2009). We consequently wish to highlight that a subset of the healthy controls (*n* = 6) was collected in EDTA (all remaining samples were collected in heparin). Furthermore, this small subset of controls was analyzed within 24 h of collection, while all other samples were analyzed within 6 h of collection (no significant differences in any of the measurements were observed between this subset and the other controls). To put into context the impairment in blood oxygen transport capacitance observed in COVID-19 subjects, we calculated the increase in cardiac output necessary to compensate for this. We would like to highlight that cardiac output was not measured in this study. In addition, we did not collect data on mechanical ventilation, which would increase pulmonary vascular resistance, due to inflation in lung volume, and would affect the cardiac output. Finally, the samples for aggregation studies were not normalized for hematocrit. Despite these limitations, our observations highlight that multiple RBC features essential for the maintenance of O_2_ delivery homeostasis and the matching of perfusion sufficiency to O_2_ demand are disrupted in hospitalized COVID-19 subjects and that the surface chemistry of the RBC may play a role in COVID-19-related RBC impairment. This could explain the enhanced risk for thromboembolic events observed in COVID-19 subjects as well as impairment to microvascular blood flow. Whilst these findings demonstrate altered RBC properties in the acute phase of infection, it would be interesting to further study and follow on hospital release to assess whether these features are prolonged for the lifespan of RBCs (i.e. 120 days) and/or play a role in long-term COVID-19.

## Data Availability

The raw data supporting the conclusion of this article will be made available by the authors, without undue reservation.
